# Evaluating the reliability of structured light entoptic tasks

**DOI:** 10.1038/s41598-026-63276-7

**Published:** 2026-07-21

**Authors:** Taranjit Singh, Mukhit Kulmaganbetov, Zhangting Wang, Dmitry A. Pushin, Benjamin Thompson, Andrew E. Silva, Melanie Mungalsingh, Iman Salehi, Davis V. Garrad, David Cory, Dusan Sarenac

**Affiliations:** 1 Centre for Eye and Vision Research (CEVR), Quantum Optics Lab, Hong Kong, PR China; 2 Entoptica Limited, Sheung Wan, Hong Kong PR China; 3 Department of Molecular Biology and Medical Genetics, Kazakh National Medical University, Almaty, 050012 Kazakhstan; 4 University of Waterloo, Institute for Quantum Computing, Waterloo, N2L 3G1 Canada; 5 Department of Physics and Astronomy, University of Waterloo, Waterloo, N2L 3G1 Canada; 6 Incoherent Vision Inc., Wellesley, N0B 2T0 Canada; 7 School of Optometry and Vision Science, University of Waterloo, Waterloo, N2L 3G1 Canada; 8 School of Optometry, The Hong Kong Polytechnic University, Hong Kong, PR China; 9 Department of Psychology, Idaho State University, Pocatello, 83209 USA; 10 Department of Chemistry, University of Waterloo, Waterloo, N2L 3G1 Canada; 11 Department of Physics, University at Buffalo, State University of New York, Buffalo, 14260 USA

**Keywords:** Diseases, Health care, Medical research, Neuroscience

## Abstract

Human perception of polarised light, known as Haidinger’s brushes, is linked to macular health but is typically too faint for reliable clinical use. Structured light can enhance these entoptic patterns, but the test-retest reliability of this approach requires validation. Here, we evaluated the reliability of a structured light-based psychophysical task in twenty-five participants (fifty eyes) across two sessions separated by one to fourteen days. Participants viewed structured light stimuli that produced rotating entoptic patterns and identified the rotation direction in a two-alternative forced-choice paradigm while a central obstruction varied in size to determine retinal eccentricity thresholds. Intraclass correlation coefficients demonstrated good to excellent reliability, with values of 0.83 for right eyes and 0.93 for left eyes. Bland-Altman analysis revealed non-significant mean differences of -0.32 degrees and − 0.12 degrees for right and left eyes, respectively, with limits of agreement spanning approximately 6° and 4° around mean thresholds of ~ 6.1° and ~ 6.5°, confirming an absence of systematic bias. These findings indicate that structured light-based psychophysical tasks can yield repeatable measurements of entoptic pattern extent in healthy individuals. Further validation in clinical populations and older age groups is required before the method can be recommended for clinical screening.

## Introduction

The assessment of visual perception, particularly in relation to macular health, has become an emerging focus within vision science^1,2^. Human perception of polarised light is enabled through Haidinger’s brushes phenomenon^3^, a visual effect that deteriorates with the progression of macular disorders^4–7^. The percept arises from the selective absorption of azimuthally polarised blue light by dichroic macular carotenoid molecules aligned with the radially oriented Henle fibres in the fovea^8–10^. The percept is inherently faint because only approximately 5–10% of macular carotenoid molecules are oriented such that they exhibit dichroic filtering^8–10^. Unfortunately, the intrinsically faint nature, transience, and low contrast of the Haidinger’s brush percept have constrained its clinical adoption, as it remains difficult to perceive reliably even in individuals with healthy vision^5–7^. Early attempts to leverage Haidinger’s brushes for clinical macular assessment demonstrated the concept but were limited by the faintness of the percept^11^.

Structured light (SL), characterised by tailored spatial and polarisation profiles, enables the generation of novel stimuli that enhance polarisation-dependent entoptic phenomena through controlled azimuthal modulation^12^. SL stimuli are emerging as a promising new method for psychophysically assessing macular health^12–18^ because they provide a larger and more readily detectable entoptic percept relative to traditional polarisation-based methods^16^. For example, SL techniques can serve as measures of circularly oriented macular pigment optical density^17,18^. This measurement may offer a functional basis for detecting early disruptions in the macula associated with diseases like age-related macular degeneration (AMD), a prospect highlighted for future clinical translation^19–21^. Other approaches to Haidinger’s brushes-based macular assessment, such as the devices developed by Temple and colleagues (Azul Optics), have also reported high reliability using enhanced polarisation stimuli^5^.

Conventional methods for assessing visual perception, such as perimetry and various visual field tests, exhibit varying levels of repeatability, influenced by factors such as individual differences in attention, cognitive load, and environmental conditions^22,23^. This variability can lead to inconsistent outcomes, raising concerns about the reliability of these assessments in both clinical and research settings^24^. Psychophysical methods for assessing macular pigment, such as heterochromatic flicker photometry (HFP), have generally demonstrated good to excellent test-retest reliability when implemented carefully^25–28^. However, these methods can be influenced by factors such as lens opacity, pupil size, and individual differences in flicker perception^28,29^. Additionally, traditional methods that rely on unaided observation of Haidinger’s brushes encounter more fundamental challenges related to the faintness and transience of the percept^3–6^.

This study aimed to evaluate the test-retest reliability of an SL-based psychophysical task that utilises SL-induced entoptic phenomena^12–18^. While the results of SL-based perception tests are influenced by macular health, they are also subjective, introducing a degree of individual variation^12,14,16–18,30^. This investigation aims to contribute to the growing body of knowledge surrounding structured light applications in vision science, paving the way for enhanced diagnostic tools that leverage the unique properties of polarised light perception.

## Methods

This cross-sectional research (ClinicalTrials.gov ID: NCT05913063, June 26, 2023) was approved by the Institutional Review Board (IRB) of The Hong Kong Polytechnic University through the Human Subjects Ethics Application Review System (HSEARS) (Reference Number: HSEARS20210910002, June 11, 2022), with the Centre for Eye and Vision Research listed as the study site. All experiments were performed in accordance with relevant guidelines and regulations, adhering to the Declaration of Helsinki. Written informed consent was obtained from all participants after they were informed of the study goals and methods.

A total of 28 participants were recruited from the Centre for Eye and Vision Research (CEVR). Every participant was subjected to inclusion screening where they underwent an eye and vision examination including assessment of medical and family history, unaided visual acuity, subjective refraction using trial frames and lenses, binocular vision tests, ocular motility, contrast sensitivity using the Thomson Chart (Thomson Software Solutions, UK), slit-lamp bio-microscopy, indirect ophthalmoscopy, colour fundus photography (Nidek AFC-330, Japan), optical coherence tomography (Topcon DRI OCT Triton, Japan), and ocular biometry (Zeiss IOL Master 700, Germany). Twenty-five participants (12 M/13F, aged 21–75 years, mean ± SD: 38.4 ± 15.2 years) with good vision (best-corrected visual acuity of 0.00 logMAR or better in each eye) and healthy eyes (with no history of eye diseases, ocular injuries, or neurological disorders) were included in the study; 3 were excluded or withdrew from the study. All participants were naïve to the structured light entoptic task, though some had general familiarity with psychophysical testing procedures by virtue of being recruited from a vision research institute.

### Psychophysical task

The task was a two-alternative forced-choice (2AFC) paradigm in which participants viewed SL-based stimuli and reported the perceived direction of rotation of the entoptic pattern (clockwise or counterclockwise).

The psychophysical task conducted in this study utilised SL-based stimuli that produced entoptic patterns composed of *N*_*f*_ = 22 brushes with a temporal frequency *ω* = 7.7 Hz (corresponding to an angular velocity 2*πω/N*_*f*_, equaling 2.19 Hz) for a duration of 0.5 s per trial^16–18^. The value of *N*_*f*_ = 22 was selected based on prior optimisation works^16,18^, which identified this brush number as providing a clearly perceptible entoptic pattern while maintaining a spatial frequency that participants could reliably resolve. The rotation frequency of 7.7 Hz was similarly chosen based on piloting that identified this rate as providing a clear sense of motion direction without exceeding temporal integration limits^16^. We note that individual differences in temporal processing may influence the optimal rotation frequency, which could represent a source of variability across participants. The laser power incident on the retina (*<* 10 nW mm^*−* 2^) remained a factor of 100 below the maximum permissible exposure for 450 nm light, ensuring no safety hazards^16,31^. The stimulus was delivered at a wavelength of 450 nm via a Maxwellian view optical system, in which the beam was focused to a point at the pupil plane, rendering the retinal image independent of the eye’s refractive state. Participants were instructed to identify the direction of rotation of the entoptic patterns, which could be either clockwise or counterclockwise, as dictated by a motorised polariser^16–18^. Before formal data collection, each participant completed 5–10 practice trials to ensure understanding of the task; these practice trials were excluded from analysis.

To assess the retinal eccentricity of their SL perception, structured-light techniques to occlude central regions of varying sizes in the generated entoptic patterns were employed. The threshold mask was created by a spatial light modulator (HOLOEYE Photonics AG, Berlin, Germany). The mask consisted of a circular central obstruction that blocked the entoptic pattern within a specified radius, while the pattern remained visible outside this region. The radius of the obstruction was adjusted according to a 2-up/1-down staircase method that has been described previously^16^. The staircase commenced with a central mask radius of 0.45^◦^ visual angle and changed with a step size of 1.35^◦^ visual angle in radius for the first three reversals, then with a step size of 0.90^◦^ for the next three reversals, then became 0.45^◦^ for the next three reversals, and finally changed to 0.23^◦^ for the remaining reversals. The staircase terminated after 14 total reversals, and the threshold was calculated as the mean of the last six reversal values. This method enables a performance accuracy measurement of 70.7% for the threshold radius of each stimulus^16^ and determines the retinal eccentricity *R*_*T*_ of the perceivable area of the SL-based stimuli. A red laser illuminated a 50 *µ*m pinhole at the centre of the pattern, producing an approximately 1^◦^ beam for participants to fixate on.

Note that to convert the radius of the central mask from spatial light modulator pixel units to visual angle degrees, a global fixed conversion ratio of 0.046 degrees per pixel, as established in a previous study^17^ to be a reliable average, was applied. This conversion rescales the data into interpretable degrees of visual angle without altering the shape or distribution of the data.

*R*_*T*_ estimates the visual angle of the observed entoptic pattern, which becomes visible due to the arrangement of radially oriented birefringent layers of photoreceptor axons and the dichroism of macular pigments located in Henle’s fibre layer of the retina, which filters blue azimuthally polarised light^16,18^. We note that the composition of macular carotenoids varies with retinal eccentricity, with zeaxanthin and meso-zeaxanthin predominating in the central fovea and lutein becoming relatively more prevalent at peripheral eccentricities^10^. Since the dichroic properties depend on the oriented arrangement of carotenoids, and carotenoid composition changes with eccentricity, the visibility of the entoptic pattern, and therefore *R*_*T*_, may reflect a complex interaction between carotenoid density, composition, and Henle fibre orientation across the measured field rather than a simple linear proxy for macular pigment spatial extent.

Participants were required to remove any refractive corrections, including contact lenses, prior to the experiment, which was conducted monocularly. As noted above, the SL stimulus was delivered via Maxwellian view and is therefore unaffected by the eye’s refractive state. However, the fixation target (red laser spot) was viewed through the eye’s natural optics, meaning that uncorrected refractive error, particularly astigmatism, could have affected the clarity of the fixation target and potentially reduced fixation stability. Both eyes were tested on the same day, with a brief interval of 1 to 2 min allowed for chinrest adjustments after the first eye was tested. The first eye examined was chosen randomly, with the other eye covered; the second eye was then tested under identical conditions.

An identical test was conducted for each participant on a separate day, with the interval between the two tests ranging from 1 to 14 days. The first eye examined was selected randomly, followed by testing the other eye under the same conditions. In total, two identical psychophysical tasks were administered to 25 participants (*n* = 50 eyes) aged 21 to 75 years.

Both participants and experimenters were aware that the second session was a repeat of the first (i.e., the retest was not conducted under blind conditions). This was unavoidable because the psychophysical procedure requires explicit subject instruction and feedback, and the technical setup was identical across sessions. However, the objective nature of the response (forced-choice discrimination of stimulus rotation) and the automated staircase procedure minimise experimenter influence.

### Statistical analysis

Two values of *R*_*T*_ were obtained from the two identical psychophysical tasks for each eye, right eye (RE) and left eye (LE). Intraclass correlation coefficients (two-way mixed-effects model) and Bland-Altman plots were used to assess test-retest reliability. In addition, the Pearson correlation coefficient between the *R*_*T*_ values in the first and second sessions was calculated to assess the correlation of results between the two sessions. Data from the left and right eyes were analysed separately^32^.

## Results

Table 1 summarises the mean and standard deviation of retinal eccentricity thresholds (*R*_*T*_) for the right and left eyes during the first and second sessions, obtained from the SL-based psychophysical task.

**Table 1:** Mean retinal eccentricity thresholds (*R*_*T*_) of all participants.

Session	Right eye (*n* = 25)		Left eye (*n* = 25)	
	1st	2^nd^	1^st^	2nd
Mean *±* SD	5.9^◦^ *±* 2.18^◦^	6.25^◦^ *±* 2.19^◦^	6.4^◦^ *±* 2.31^◦^	6.54^◦^ *±* 2.11^◦^

The interpretation of intraclass correlation coefficient (ICC) values is critical for assessing measurement reliability, especially for measurements from a response-based task. As a general guideline, ICC values above 0.75 suggest good to excellent reliability, indicating that the measurements are highly reliable and can be used confidently in clinical or research settings^23,33–35^. The test-retest reliability was calculated as the ICC using a two-way mixed-effects, single-rater model (ICC(3,1)). The calculation was based on the following formulation (1), which quantifies the correlation between two test sessions:

1 $$\:r=\frac{1}{N{s}^{2}}\sum\:_{n=1}^{N}({x}_{n,1}-\stackrel{\prime }{x}\left)\right({x}_{n,2}-\stackrel{\prime }{x})$$

where *N* is the number of subjects, *s*^2^ is the pooled variance across both sessions, *x*_*n*,1_ and *x*_*n*,2_ are the measurements for the.

*n*-th subject in session 1 and session 2, respectively, and *x*¯ is the overall mean across all measurements from both sessions.

ICCs were 0.83 [95% CI: 0.62–0.93] for right eyes (RE) and 0.93 [95% CI: 0.84–0.97] for left eyes (LE) (Table 2), indicating good to excellent test-retest reliability for each eye^23,33–35^.

**Table 2:** Two-way mixed-effects intraclass correlation coefficient values of individual eyes.

	Right eye (*n* = 25)	Left eye (*n* = 25)
ICC [95% CI]	0.83 [0.62, 0.93]	0.93 [0.84, 0.97]

The Bland-Altman analysis (Table 3) revealed a non-significant mean difference of *−* 0.32^◦^ (SD: 1.51) and *−* 0.12^◦^ (SD: 1.03) for RE and LE, respectively, between the first and second sessions, with limits of agreement ranging from *−* 3.28^◦^ to.

**Table 3:** Bland-Altman analysis of individual eyes.

	Right eye (*n* = 25)	Left eye (*n* = 25)
Mean difference ± SD	−0.32^◦^ ± 1.51^◦^	−0.12^◦^ ± 1.03^◦^
Limits of agreement	*−*3.28^◦^ to 2.64^◦^	*−*2.14^◦^ to 1.89^◦^

**Fig. 1:**
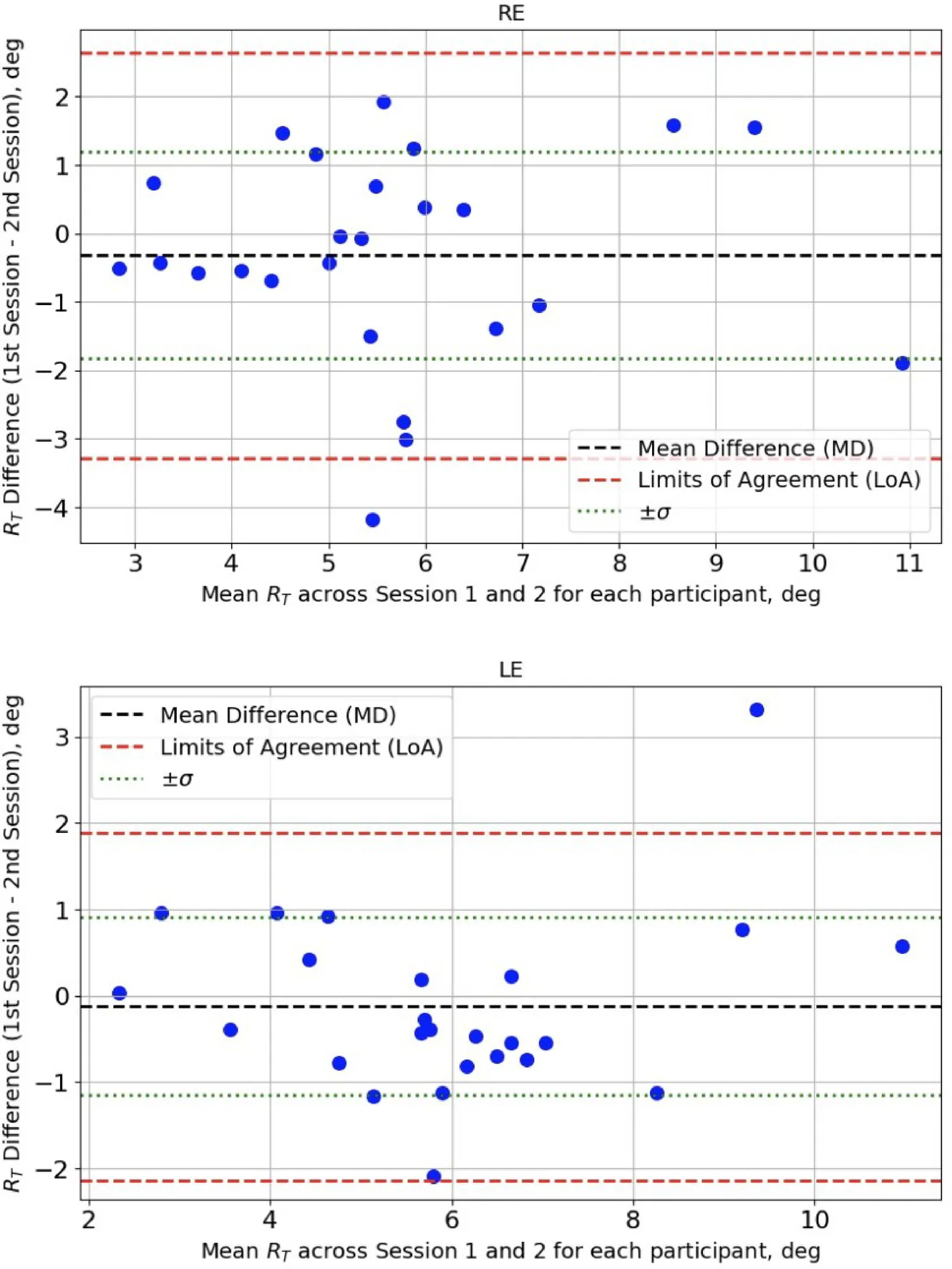
Bland-Altman plot with mean *R*_*T*_ across Session 1 and 2 against the difference of *R*_*T*_ over the two sessions for each participant, for the right eye (RE, top) and left eye (LE, bottom). There was nearly zero bias between the 1 st and 2nd sessions for both eyes. The mean RT (averaged across sessions) was approximately 6.1° for RE and 6.5° for LE, providing context for the width of the limits of agreement.

**Fig. 2:**
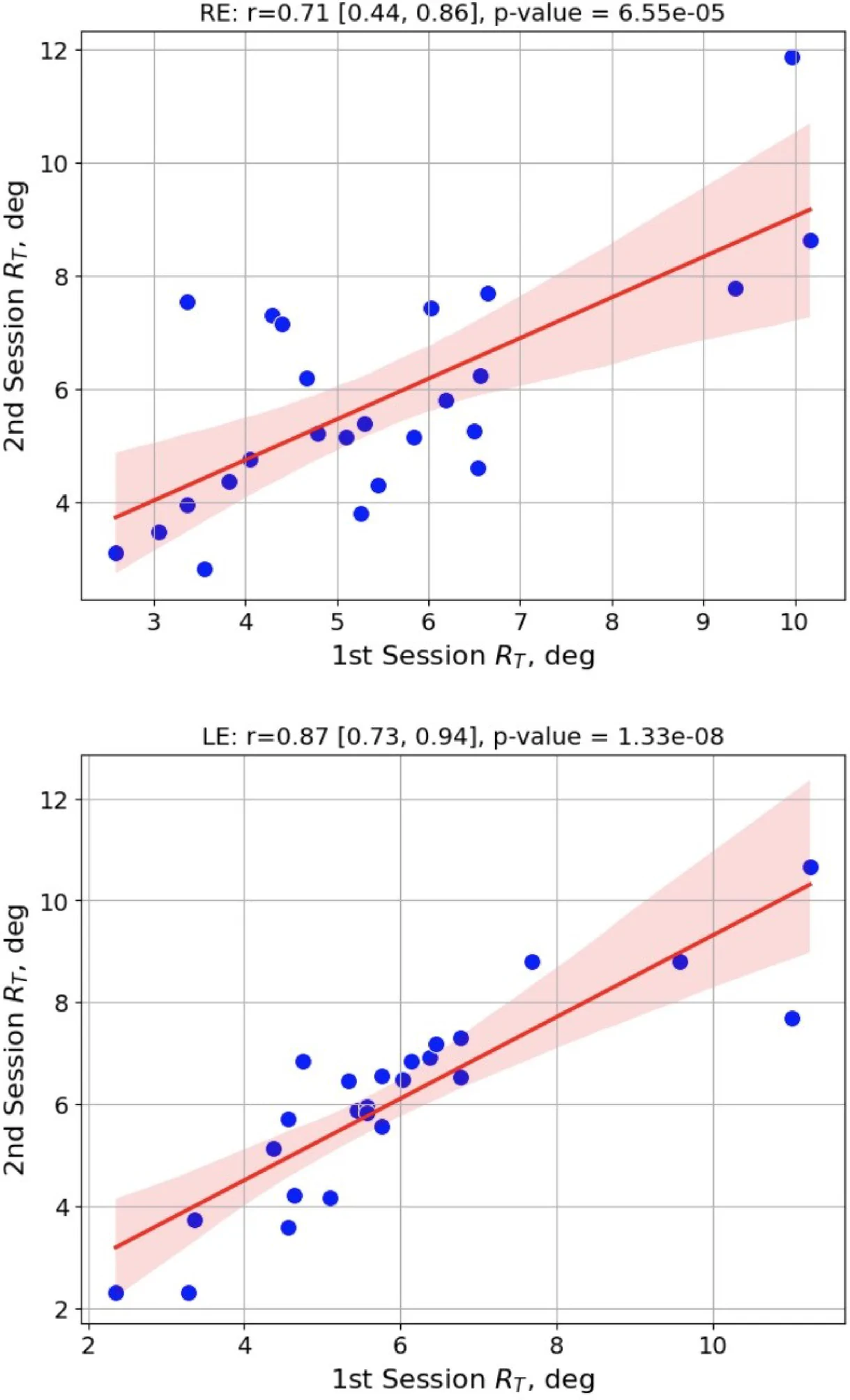
Linear correlation plot between *R*_*T*_ values of the first and second sessions. It shows a Pearson correlation coefficient (*r*), confidence interval and p-value of *R*_*T*_ for the right eye (RE, top) and left eye (LE, bottom), indicating a strong association of results for both eyes between the two sessions.

2.64^◦^ and *−* 2.14^◦^ to 1.89^◦^ (Fig. 1) for RE and LE, respectively. Given the mean *R*_*T*_ values of approximately 6.1° (RE) and 6.5° (LE), the limits of agreement represent a range of approximately ± 3° around the mean, corresponding to roughly ± 50% of the mean threshold value. In addition, the Pearson correlation coefficient between the *R*_*T*_ value in the first and second sessions was 0.71 for RE and 0.87 for LE, respectively, with a *p*-value *<* 0.01 for both eyes (Fig. 2), indicating good correlation of results between the two sessions. We note that three eyes (from two participants, aged 23 and 26 years) had *R*_*T*_ values exceeding 9.5° in at least one session. These outlying values likely reflect the inherent variability of subjective psychophysical threshold measurement rather than genuinely larger macular pigment distributions, though the enhanced sensitivity of the SL technique to faint peripheral entoptic signals may also contribute.

## Discussion

When interpreting the ICC values for test-retest reliability, it is essential to recognise that different studies and fields may adopt varying guidelines. While no universally accepted standard exists, several common interpretations have emerged in the literature. For instance, Koo and Li (2016)^35^ propose that ICC values below 0.50 indicate poor reliability, those between.

0.50 and 0.75 suggest moderate reliability, values from 0.75 to 0.90 indicate good reliability, and values above 0.90 signify excellent reliability. In contrast, Cicchetti (1994)^35^ presents a slightly different framework, categorising values under 0.40 as poor, between 0.40 and 0.59 as fair, between 0.60 and 0.74 as good, and between 0.75 and 1.00 as excellent. These varying interpretations underscore the necessity of careful consideration of context and application when evaluating reliability based on ICC values^35^.

Building on this foundation, an average ICC value of 0.88 (RE = 0.83, LE = 0.93) in this study can be classified as reflecting good to excellent reliability^36^ his finding indicates that the SL-based psychophysical method is a reliable approach for testing polarisation perception in the human eye. This reliability is further corroborated by the Pearson correlation coefficients, which demonstrated a strong positive association between the first and second sessions for both the right (*r* = 0.71, *p* < 0.01) and left eyes (*r* = 0.87, *p* < 0.01). Additionally, Bland-Altman analysis indicated a negligible mean difference between sessions (*−* 0.32^◦^ for RE and *−* 0.12^◦^ for LE), confirming the absence of systematic bias or learning effects across the testing interval. However, the limits of agreement (spanning approximately 6° for RE and 4° for LE) indicate that session-to-session variability of roughly ± 3° should be expected for individual measurements. This level of fluctuation is consistent with the inherent challenges of subjective psychophysical tasks involving the detection of faint entoptic thresholds. The combination of high ICC, strong correlation, and negligible bias suggests that the method provides consistent measurements suitable for assessing macular function in healthy individuals, though the individual-level variability should be considered when interpreting longitudinal changes.

However, it is important to address potential limitations that may arise from this method. Specifically, the first test, even when administered identically, could influence the results of subsequent tests. Given the psychophysical nature of the task, there is a possibility of a natural learning effect that may enhance participants’ performance during the second test administration, potentially resulting in a higher *R*_*T*_ value.

One notable study employed a similar psychophysical task and investigated the effects of eye dominance and testing order on the resulting thresholds^18^. A random eye was selected to perform the psychophysical task first, followed immediately by the other eye, and their analysis revealed no statistically significant difference between the first and second eye tested. In the current study, the mean difference between the first and second sessions was minimal, at *−* 0.22^◦^, with approximately 40% of participants demonstrating a lower *R*_*T*_ value in the second session compared to the first. These findings suggest that learning did not influence the outcomes of this psychophysical task and its test-retest reliability^18,23^.

A key limitation of this study is that all participants had normal vision, and the sample was not stratified by age. While the age range was broad (21–75 years), a supplementary analysis revealed no significant correlation between age and mean *R*_*T*_ (*r* = 0.18, *p* = 0.39), likely due to the relatively small and unbalanced sample. A dedicated study with balanced age groups is needed to determine whether age-related changes in macular pigment influence the reliability of the SL-based task.

Another important limitation is the absence of a concurrent measure of macular pigment optical density (MPOD). Without MPOD data, we cannot directly assess the convergent validity of *R*_*T*_ as a functional measure of macular pigment distribution, nor can we determine the extent to which individual differences in MPOD contribute to the observed variability in *R*_*T*_. Future studies should co-measure MPOD using established techniques such as HFP or reflectometry to address this gap.

Additionally, while the SL stimulus itself was delivered via Maxwellian view and is therefore unaffected by refractive error, the fixation target was viewed through the eye’s natural optics. Uncorrected refractive error, particularly astigmatism, may have reduced fixation stability in some participants, potentially contributing to session-to-session variability. Future implementations should consider correcting refractive error for the fixation target or employing an improved fixation monitoring system.

A limitation of this study is that all participants had normal vision. Further work is required to assess whether individuals with early-stage macular disease exhibit similar test-retest reliability. Furthermore, the choice of stimulus parameters (brush number and rotation frequency), while based on prior optimisation, may not be optimal for all individuals, particularly those with reduced temporal processing abilities or altered macular pigment composition. Systematic parameter optimisation in diverse populations represents an important avenue for future work.

## Conclusions

These findings indicate that SL-based psychophysical tasks can yield repeatable measurements of entoptic pattern extent in healthy individuals, with negligible systematic bias between sessions. However, the limits of agreement suggest that individual-level measurements carry a degree of variability that should be considered when interpreting longitudinal changes. Further optimisation of the protocol, co-validation with established MPOD measures, and validation in clinical populations and older age groups are needed to establish the method’s suitability for clinical screening of diseases affecting macular health.

## Data Availability

Data available on request from the authors.
